# The impact of dietary copper intake on cardiovascular morbidity and mortality among hypertensive patients: a longitudinal analysis from NHANES (2001–2018)

**DOI:** 10.1186/s12889-025-22178-5

**Published:** 2025-03-10

**Authors:** Haibin Xu, Zhou Liu, Baohong Yao, Ziqi Xu

**Affiliations:** 1 Cardiovascular Medicine, First affiliated Hospital of Huzhou University, Huzhou, 313000 China; 2 General Medicine, First affiliated Hospital of Huzhou University, Huzhou, 313000 China; 3 Graduate School, Lanzhou University, No. 222 Tianshui South Road, Lanzhou City, China; 4 Department of Orthopedics, The First People’s Hospital of Lin’an District, No. 360 Yikang Street, Jinnan Street, Lin’an District, Hangzhou, China

**Keywords:** Dietary copper intake, Cardiovascular diseases, Mortality, NHANES

## Abstract

While the impact of macronutrients on health is well-understood, the influence of micronutrients such as copper on cardiovascular health remains less explored. Copper, vital for heart function and antioxidant defense, may affect cardiovascular health through its role in enzymatic activities that reduce oxidative stress. This study evaluates the relationship between dietary copper intake and cardiovascular outcomes in hypertensive patients using data from the National Health and Nutrition Examination Survey (NHANES) from 2001 to 2018. Findings reveal that higher dietary copper intake is associated with significantly lower cardiovascular disease (CVD) prevalence and mortality rates. A non-linear relationship was identified, indicating an optimal copper intake threshold of approximately 2.85 mg/day. Notably, the protective effects of copper were more pronounced in men, non-diabetic individuals, and those with higher educational levels. These results underscore copper’s potential role in preventing cardiovascular complications in hypertensive patients and support the inclusion of copper intake in dietary recommendations to improve cardiovascular health. This study enhances our understanding of how micronutrients influence cardiovascular disease management and aids in developing targeted nutritional interventions.

## Introduction

Hypertension is a predominant risk factor for CVDs, which are the leading cause of mortality worldwide [1]. The global burden of these diseases continues to rise, influencing public health policies and healthcare practices across diverse populations [2]. Epidemiologically, hypertension affects over a billion people globally and is directly linked to approximately half of all heart disease and stroke cases [3]. Alongside genetic, environmental, and lifestyle factors, dietary components play a crucial role in the modulation of blood pressure and overall cardiovascular risk. While the impact of macronutrients has been extensively studied, the influence of micronutrients, particularly trace minerals like copper, is not as well-documented but is increasingly recognized as significant in maintaining cardiovascular health [4].

Copper, a trace mineral found in foods such as shellfish, nuts, seeds, and whole grains, is essential for numerous bodily functions, including angiogenesis, heart muscle contraction, and antioxidant defense [5]. Despite its critical roles, the specific mechanisms by which copper intake influences cardiovascular health remain under-explored [6]. Copper contributes to the function of important enzymes like superoxide dismutase, which protects cells from oxidative damage, and plays a role in maintaining endothelial and myocardial health [7]. Preliminary studies suggest that copper deficiency may be associated with increased heart disease risk due to its role in maintaining myocardial tissue integrity and vascular elasticity [6]. However, gaps remain in understanding the optimal intake levels and the direct effects of copper on cardiovascular morbidity and mortality, particularly among those already at risk, such as hypertensive patients [8].

Previous studies exploring the association between copper and CVDs often suffer from limitations like small sample sizes and cross-sectional designs [7]. The primary objective of this study is to explore the relationship between dietary copper intake and cardiovascular outcomes in hypertensive patients [9]. We aim to quantify copper intake among hypertensive individuals and investigate its association with the incidence of major cardiovascular events, including myocardial infarctions and strokes [7]. Additionally, the study will adjust for confounders like age and gender to delineate any dose–response relationships [10]. The results are expected to inform dietary recommendations and potentially influence public health guidelines for reducing cardiovascular risk in this population by incorporating copper intake considerations into nutritional recommendations and hypertension management strategies [11].

## Methods

### Data collection

We utilized cross-sectional data from the National Health and Nutrition Examination Survey (NHANES), which is specifically designed to reflect the health and nutritional status of the entire U.S. population. NHANES is distinctive in its methodology, combining both interviews and physical examinations to gather comprehensive data. The survey covers a wide range of information, including demographic, socioeconomic, dietary, and health-related questions. Our research exclusively uses data that is publicly available and does not require additional ethical approval, adhering to the standards set by the NCHS Ethics Review Board (ERB). For more information on ERB approval, please visit https://www.cdc.gov/nchs/nhanes/irba98.htm. The dataset can be accessed freely at the Centers for Disease Control and Prevention (CDC) website: https://wwwn.cdc.gov/nchs/nhanes/Default.aspx.

**Fig. 1:**
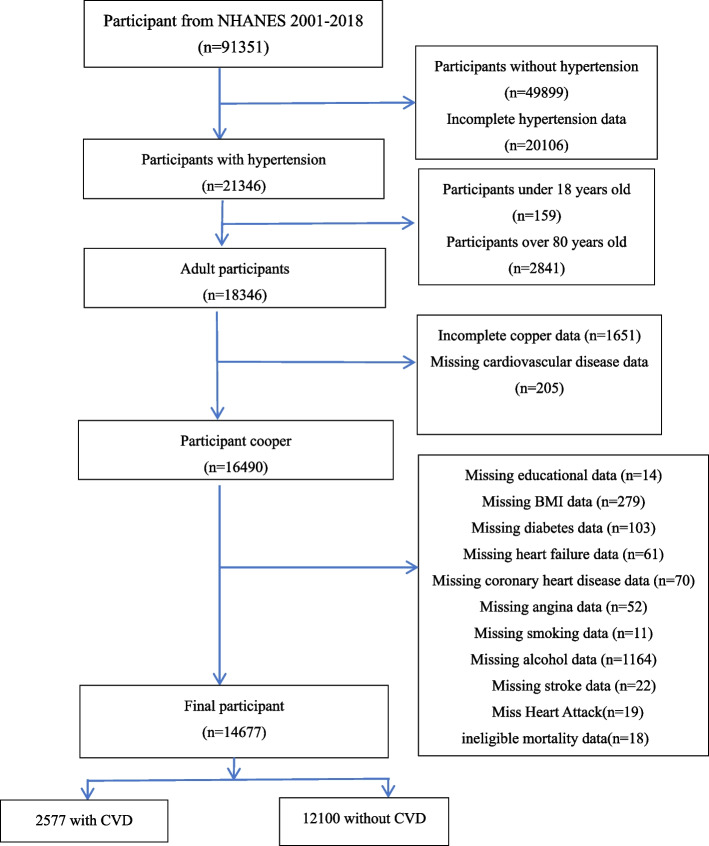
The flowchart of study sample selection form NHANES 2001–2018

This study utilized data from nine NHANES cycles (2001–2018), a nationally representative survey conducted by the National Center for Health Statistics (NCHS). NHANES employs a complex, multi-stage probability sampling method to ensure that the collected data reflect the health and nutritional status of the U.S. non-institutionalized civilian population (NCHS, 2023) [12]. The survey integrates structured interviews, physical examinations, and laboratory tests, making it a valuable resource for epidemiological studies. Dietary copper intake was assessed using two 24-hour dietary recall (24HR) interviews, a validated method widely used in large-scale nutritional studies (CDC, 2023) [13]. The first recall was conducted in-person at the NHANES Mobile Examination Center (MEC) by trained interviewers using the Automated Multiple-Pass Method (AMPM), a standardized approach developed by the U.S. Department of Agriculture (USDA) to improve dietary recall accuracy (USDA, 2023) [14]. The second recall was conducted via telephone 3–10 days later to account for intra-individual day-to-day dietary variability. Copper intake values (mg/day) were estimated by linking reported food and beverage consumption to the USDA Food Composition Database, which provides standardized nutrient values for various foods (USDA, 2023) [15]. The average of both recalls was used to enhance measurement reliability. Participants with only one recall were excluded, as single-day recalls do not adequately represent habitual dietary intake and may introduce measurement error. Initially, 91,351 participants were identified across the nine NHANES cycles, but after applying inclusion and exclusion criteria, a final sample of 14,677 hypertensive participants was included. The inclusion criteria were: (1) adults aged 18–80 years and (2) a diagnosis of hypertension, defined as systolic blood pressure (SBP) ≥140 mmHg or diastolic blood pressure (DBP) ≥90 mmHg, self-reported hypertension, or current use of antihypertensive medication, in accordance with the International Society of Hypertension (ISH) guidelines. The exclusion criteria included (1) participants lacking dietary copper intake data (i.e., those who did not complete both 24HR recalls), (2) individuals with missing demographic or health-related covariate data, including age, sex, BMI, smoking status, alcohol use, diabetes status, and cardiovascular outcomes, (3) participants outside the defined age range (younger than 18 or older than 80 years), and (4) individuals with incomplete cardiovascular disease (CVD) outcome data. Given the complex sampling design of NHANES, survey weights, strata, and primary sampling units (PSUs) were incorporated into all statistical analyses to ensure that the findings were nationally representative of the U.S. adult hypertensive population and to minimize potential biases arising from differential selection probabilities and non-response rates (CDC, 2023) [16]. A detailed flowchart illustrating the sample selection process is presented in Figure 1.

### Exposure measurement methods

In the NHANES database, dietary copper intake was assessed using two 24-hour dietary recall interviews. Participants completed the first interview in person at the NHANES Mobile Examination Center, and the second was conducted via telephone 3 to 10 days later. For this study, dietary copper intake was calculated by averaging the data from these two recalls, and cases with only a single recall were excluded. The dietary data were collected through nutrition questionnaires and 24-hour dietary recall surveys, where participants reported all food and beverage consumption over the past one to two days. These data were then matched with the USDA Food Composition Database to estimate the copper content of the reported foods. To ensure accuracy and representativeness, researchers averaged dietary data over multiple days and accounted for individual differences, including the use of dietary supplements [17].

### Outcome variable selections

In this study, the primary outcomes were cardiovascular diseases (CVDs) and mortality. CVDs were identified through either reported or self-admitted physician diagnoses. We assessed self-reported CVD by asking participants whether a doctor or other health professional had ever informed them that they had suffered from heart-related conditions such as a heart attack, coronary heart disease, angina, congestive heart failure, or stroke. The response options provided were "Yes," "No," or "Don't know," with "Yes" indicating a diagnosis of CVD. Participants who responded "Don't know" were excluded from the analysis.For mortality data, we utilized the NHANES Public-Use Linked Mortality Files available up to December 31, 2019, which can be found at CDC's website. These files are linked to the National Death Index (NDI) data using probabilistic matching algorithms to ascertain mortality status. Mortality outcomes were classified according to the International Classification of Diseases, 10th Revision (ICD-10). In ICD-10, cardiovascular-related deaths are identified by specific codes, such as I50 for congestive heart failure and I60-I69 for stroke or cerebrovascular accidents. The follow-up period for each participant extended from the baseline examination date to the date of last known alive status or the date of removal from the mortality archive due to death.

### Covariates extraction

Covariates that could influence the relationship between dietary copper intake and the risk of cardiovascular disease (CVD) or CVD-related mortality in hypertensive patients were collected through interviews and medical examinations. These include sociodemographic and lifestyle factors like age, gender, race/ethnicity, education level, and body mass index (BMI). Demographic data on age, gender, race (Non-Hispanic White, Non-Hispanic Black, Mexican American, and other races), and education level (below high school, high school, university, or higher) were gathered through interviews.

## Data analysis

Data analysis was performed using R software (version 4.3.0). We adopted NHANES-specific weighting procedures to ensure that our estimates are representative of the U.S. population, accounting for selection biases and non-response. Each participant sampled from the 2001–2018 datasets was assigned a weight appropriate for the combined survey cycles.For the association between dietary copper intake and the prevalence of cardiovascular diseases (CVD) among hypertensive patients, we used weighted multivariate logistic regression to estimate odds ratios (ORs) and 95% confidence intervals (CIs). Dietary copper intake was divided into quartiles, with the lowest quartile (Q1) serving as the reference group. Three models were implemented to refine our understanding of this relationship: Model 1 remained unadjusted; Model 2 was adjusted for demographic factors such as age, sex, race, and education level; and Model 3 was further adjusted for clinical and lifestyle factors including body mass index (BMI), smoking and drinking status, and diabetes.We also employed Cox proportional hazards regression models to estimate hazard ratios (HRs) for all-cause and CVD-specific mortality. These models accounted for censoring and the time-dependent nature of risk, structured similarly across three models: an unadjusted Model 1, a demographically adjusted Model 2, and a fully adjusted Model 3 incorporating both demographic and clinical variables.To explore potential nonlinear relationships between dietary copper intake and health outcomes, a restricted cubic spline (RCS) model was applied. This analysis helped to identify any thresholds beyond which the benefits of copper intake on mortality rates may plateau or alter significantly.All statistical analyses were two-sided, and a significance level was maintained at *p* < 0.05. This rigorous statistical approach enabled us to draw robust conclusions about the dose–response relationship between copper intake and cardiovascular outcomes, informing subsequent clinical and dietary recommendations.

## Results

### Characteristics of the study population

The characteristics of the study population are summarized in Table 1, highlighting differences between individuals with and without cardiovascular disease (CVD). Individuals with CVD are, on average, older (62.46 ± 0.30 years vs. 53.29 ± 0.21 years, *P* < 0.0001), have a lower mean dietary copper intake (1.17 ± 0.02 mg/day vs. 1.27 ± 0.01 mg/day, *P* < 0.0001), a higher BMI (31.75 ± 0.20 vs. 31.10 ± 0.10, P = 0.002), and are more likely to be male (54.05% vs. 49.92%, P = 0.002). Ethnically, White individuals have the highest prevalence of CVD (71.52%), followed by Black (14.94%), Mexican (7.19%), and other ethnic groups (9.42%) (P = 0.01). Higher education is associated with lower CVD prevalence, with college graduates having a prevalence of 16.13% compared to higher rates among less educated individuals (*P* < 0.0001). Diabetes mellitus (DM) is strongly associated with CVD, with 42.01% of individuals with CVD having DM compared to 20.83% of those without (*P* < 0.0001). Smoking patterns show that 39.91% of individuals with CVD are former smokers, 24.52% are current smokers, and 35.57% have never smoked (*P* < 0.0001). Alcohol consumption patterns differ significantly, with heavy drinking more prevalent among non-CVD individuals (18.61% vs. 13.01%, *P* < 0.0001), whereas former alcohol users are more common in the CVD group (29.28% vs. 16.21%). Additionally, individuals with CVD have significantly higher rates of other cardiovascular conditions, such as stroke (33.29% vs. 0%), angina (29.24% vs. 0%), coronary heart disease (40.72% vs. 0%), congestive heart failure (28.23% vs. 0%), and heart attack (39.70% vs. 0%) (*P* < 0.0001 for all comparisons). Regarding dietary copper intake, higher quartiles are linked to lower CVD prevalence, with individuals in the highest quartile (Q4: 1.46–46.24 mg/day) having the lowest prevalence of CVD (23.74%), and those in the lowest quartile (Q1: 0.00–0.754 mg/day) having the highest prevalence (25.64%) (*P* < 0.0001). These findings underscore significant associations between dietary copper intake, demographic factors, lifestyle behaviors, and CVD morbidity and mortality (See Table 1).

**Table 1:** The general characteristics of the population in NHANESE 2001–2018

Variable	Total	No-CVD	CVD	*P*
**Age**	54.66 ± 0.20	53.29 ± 0.21	62.46 ± 0.30	< 0.0001
**Copper (mg/day)**	1.26 ± 0.01	1.27 ± 0.01	1.17 ± 0.02	< 0.0001
**BMI**	31.20 ± 0.09	31.10 ± 0.10	31.75 ± 0.20	0.002
**Sex**				0.002
Female	7255(49.46)	6119(50.08)	1136(45.95)	
Male	7422(50.54)	5981(49.92)	1441(54.05)	
**Ethic**				0.01
Black	4051(13.87)	3317(13.68)	734(14.94)	
Mexican	2078 (5.69)	1795(5.97)	283(4.12)	
Other	2217 (9.93)	1893(10.02)	324 (9.42)	
White	6331 (70.51)	5095(70.33)	1236(71.52)	
**Education**				< 0.0001
Less than 9th grade	1808 (6.01)	1422(5.47)	386(9.07)	
9-11th grade (Includes 12th grade with no diploma)	2275(11.57)	1779(10.92)	496(15.28)	
High school graduate/GED or equivalent	3591(25.68)	2940(25.26)	651(28.04)	
Some college or AA degree	4233(32.14)	3502(32.25)	731(31.48)	
College graduate or above	2770(24.61)	2457(26.10)	313(16.13)	
**Diabetes**				< 0.0001
DM	4328(24.00)	3152(20.83)	1176(42.01)	
IFG	814 (6.28)	676(6.17)	138(6.92)	
IGT	521 (3.41)	438(3.48)	83(3.00)	
No	9014(66.31)	7834(69.53)	1180(48.07)	
**Smoke**				< 0.0001
Former	4382(30.68)	3367(29.05)	1015(39.91)	
Never	7217(48.98)	6281(51.35)	936(35.57)	
Now	3078(20.34)	2452(19.61)	626(24.52)	
**Alcohol user**				< 0.0001
Former	3176(18.17)	2341(16.21)	835(29.28)	
Heavy	2441(17.77)	2130(18.61)	311(13.01)	
Mild	4971(37.54)	4155(37.93)	816(35.33)	
Moderate	1978(15.40)	1732(16.26)	246(10.56)	
Never	2111(11.11)	1742(10.99)	369(11.82)	
**Stroke**				< 0.0001
No	13748(95.01)	12100(100.00)	1648 (66.71)	
Yes	929 (4.99)	0 (0.00)	929(33.29)	
**Coronary heart disease**				< 0.0001
No	13719(93.89)	12100(100.00)	1619 (59.28)	
Yes	958 (6.11)	0 (0.00)	958(40.72)	
**Angina**				< 0.0001
No	14013(95.61)	12100(100.00)	1913 (70.76)	
Yes	664 (4.39)	0(0.00)	664(29.24)	
**Congestive heart failure**				< 0.0001
No	13903(95.77)	12100(100.00)	1803 (71.77)	
Yes	774 (4.23)	0 (0.00)	774(28.23)	
**Heart attack**				< 0.0001
No	13667(94.05)	12100(100.00)	1567 (60.30)	
Yes	1010 (5.95)	0 (0.00)	1010(39.70)	
**CopperQ (mg/day)**				< 0.0001
**Q1** [0,0.754]	3509(20.42)	2758(19.53)	751(25.46)	
**Q2**(0.75,1.06]	3586(23.64)	2947(23.45)	639(24.67)	
**Q3**(1.06,1.46]	3758(27.09)	3129(27.26)	629(26.13)	
**Q4**(1.46,46.24]	3824(28.85)	3266(29.75)	558(23.74)	

Percentages are calculated as column percentages based on the total number of participants in each column. *P*-values are derived from chi-square tests for categorical variables and t-tests for continuous variables. *DM* Diabetes Mellitus, *IFG* Impaired Fasting Glucose, *IGT* Impaired Glucose Tolerance. Copper intake was divided into quartiles based on the distribution among participants. The first quartile (Q1) ranged from 0 to 0.754 mg/day, while the fourth quartile (Q4) ranged from 1.46 to 46.24 mg/day

### Association between dietary copper intake and the prevalence of cardiovascular disease in the hypertensive population

The table 2 presents the association between dietary copper intake and the prevalence of cardiovascular disease (CVD) in the hypertensive population, evaluated through three different models using multivariable logistic regression. In Model 1 (unadjusted), higher quartiles of copper intake (Q2, Q3, Q4) were significantly associated with reduced CVD prevalence compared to the reference group, Q1. For instance, Q4 exhibited a markedly lower prevalence of CVD with a coefficient of -0.49 (95% CI: 0.53 to 0.71, *P* < 0.0001), indicating a protective association. Model 2, adjusted for age, sex, race, and education level, confirmed this trend, with Q4 showing a coefficient of -0.34 (95% CI: 0.61 to 0.84, *P* < 0.0001). Finally, Model 3, which further accounted for BMI, smoking and drinking status, and diabetes, consistently demonstrated that Q4 maintained a significantly lower CVD prevalence than Q1, with a coefficient of -0.27 (95% CI: 0.65 to 0.91, *P* = 0.002). This trend across the models, with all P values for trend below 0.01, reveals a robust and dose-responsive association between increasing dietary copper intake and decreased CVD prevalence in hypertensive individuals, even after adjusting for various confounding factors (See Table 2).

**Table 2:** Association between dietary copper intake and CVD prevalence

Copper	Model 1		Model 2		Model 3	
	*B(95%CI)*	*P*	*B(95%CI)*	*P*	*B(95%CI)*	*P*
Q1	*ref*		*ref*		*ref*	
Q2	-0.21(0.69,0.94)	0.01	-0.19(0.70,0.97)	0.02	-0.17(0.71,0.99)	0.04
Q3	-0.31(0.62,0.88)	< 0.001	-0.26(0.64,0.93)	0.001	-0.21(0.67,0.98)	0.03
Q4	-0.49(0.53,0.71)	< 0.0001	-0.34(0.61,0.84)	< 0.0001	-0.27(0.65,0.91)	0.002
*P* for trend	< 0.0001		< 0.001		0.003	

Model 1 was unadjusted

Model 2 was adjusted for age, sex, race, and education level

Model 3 was further adjusted for BMI, smoking and drinking status, and diabetes

### Establish cox proportional hazard model

The table 3 shows the relationship between different dietary copper intake quartiles (Q1-Q4) and all-cause mortality. Q1 (the lowest quartile of copper intake) serves as the reference group. In Model 1 (unadjusted), the hazard ratios (HRs) for Q2, Q3, and Q4 are 0.82 (*P* = 0.01), 0.65 (*P* < 0.0001), and 0.53 (*P* < 0.0001), respectively, indicating a progressive decline in all-cause mortality risk with increased copper intake. Model 2, adjusted for age, sex, race, and education level, provides HRs of 0.86 (*P* = 0.05), 0.71 (*P* < 0.0001), and 0.68 (*P* < 0.0001) for Q2, Q3, and Q4. In Model 3, further adjusted for BMI, smoking, drinking status, and diabetes, the HRs for Q2, Q3, and Q4 are 0.90, 0.77, and 0.76, respectively. This consistent trend across models shows that individuals with higher dietary copper intake have a significantly lower risk of all-cause mortality than those with lower intake (See Table 3).

**Table 3:** Mortality outcomes by dietary copper intake (Quartiles) among participants during the follow-up period

	The quartile of Dietary copper intake					
	Q1(0–0.742)		Q2(0.742–1.038)	Q3(1.038–1.438)	Q4(1.438,46.237)	*P* for trend
**All-Cause Mortality**						
Number of deaths	773	675		638	548	
Model 1 h (*95% CI*) *P*	1	0.82(0.70,0.95) 0.01		0.65(0.57,0.73) < 0.0001	0.53(0.46,0.61) < 0.0001	< 0.0001
Model 2 h* (95% CI) P*	1	0.86(0.74,1.00) 0.05		0.71(0.62,0.81) < 0.0001	0.68(0.59,0.79) < 0.0001	< 0.0001
Model 3 h* (95% CI) P*	1	0.90(0.77,1.05) 0.19		0.77(0.67,0.88) < 0.001	0.76(0.65,0.89) < 0.001	< 0.0001
**CVD Mortality**						
Number of deaths	251	238		230	169	
Model 1 h* (95% CI) P*	1	0.84(0.66,1.06) 0.14		0.69(0.56,0.85) < 0.001	0.48(0.38,0.61) < 0.0001	0.004
Model 2 h* (95% CI) P*	1	0.88(0.68,1.13) 0.31		0.75(0.59,0.95) 0.02	0.66(0.52,0.84) < 0.001	< 0.0001
Model 3 h* (95% CI) P*	1	0.93(0.73,1.19) 0.57		0.81(0.64,1.03) 0.08	0.75(0.58,0.96) 0.02	0.013

Model 1 was unadjusted

Model 2 was adjusted for age, sex, race, and education level

Model 3 was further adjusted for BMI, smoking and drinking status, and diabetes

For cardiovascular disease (CVD) mortality, Model 1 (unadjusted) shows that the HR for Q2 is 0.84 (*P* = 0.14), which is not statistically significant. However, Q3 and Q4 show significantly reduced CVD mortality risks, with HRs of 0.69 (*P* < 0.001) and 0.48 (*P* = 0.004), respectively. In Model 2, adjusted for age, sex, race, and education, the HRs for Q2, Q3, and Q4 are 0.88, 0.75 (*P* = 0.02), and 0.66 (*P* < 0.001). After further adjustments in Model 3 for BMI, smoking, drinking status, and diabetes, Q3 and Q4 still show HRs of 0.81 (*P* = 0.08) and 0.75 (*P* = 0.02). These results indicate a correlation between higher dietary copper intake and reduced CVD mortality, suggesting that higher copper intake may offer protective benefits against cardiovascular mortality (See Table 3).

### Nonlinear relationship detection

**Fig. 2:**
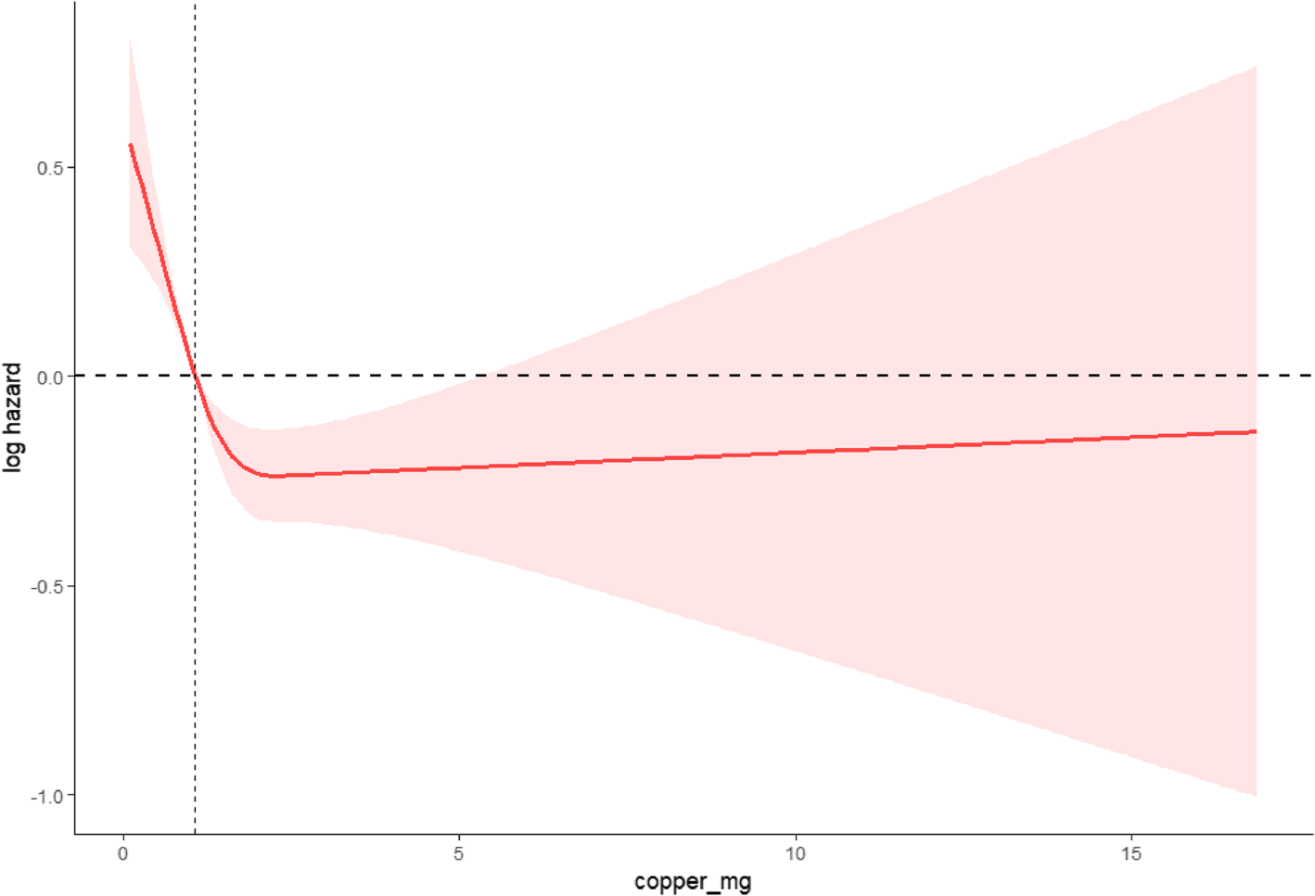
Impact of dietary copper intake on all-cause mortality risk in hypertensive patients: a restricted cubic spline analysis

The Restricted Cubic Spline (RCS) analysis of dietary copper intake and its impact on all-cause mortality among hypertensive patients provides insightful data on the non-linear relationship between copper intake and mortality risks. The RCS curve, derived from a comprehensive R language analysis incorporating adjustments for age, sex, race, education level, BMI, smoking, and drinking status, as well as diabetes, illustrates key points in the effect of copper intake on mortality. From the RCS curve, we observe an initial sharp decrease in the log hazard of all-cause mortality as copper intake increases from the lowest levels up to about 2.85 mg/day, indicating a strong protective effect of moderate copper intake against mortality. This decreases levels off and the curve stabilizes beyond this intake amount, suggesting diminishing returns in mortality reduction with higher levels of copper intake. The plot shows that increasing copper intake beyond approximately 5 mg/day does not provide additional benefits and might even potentially increase mortality risk slightly, although the curve remains relatively stable in this higher intake range. The analysis identifies a critical inflection point at a copper intake of 2.85 mg/day, where the log hazard of mortality begins to stabilize. This point is statistically significant and is crucial for understanding the optimal range of dietary copper intake that could confer the most significant health benefit (see figure 2).

It is evident that while increasing copper intake is generally beneficial up to a certain point, excessively high intakes do not confer additional protective effects and could be unnecessary or potentially harmful. This RCS analysis thus emphasizes the importance of balancing copper intake within a specific range to optimize health outcomes for hypertensive individuals. The findings strongly support the inclusion of copper intake considerations in dietary guidelines for this population, aiming to harness its benefits while avoiding potential risks associated with excessive consumption.

**Fig. 3:**
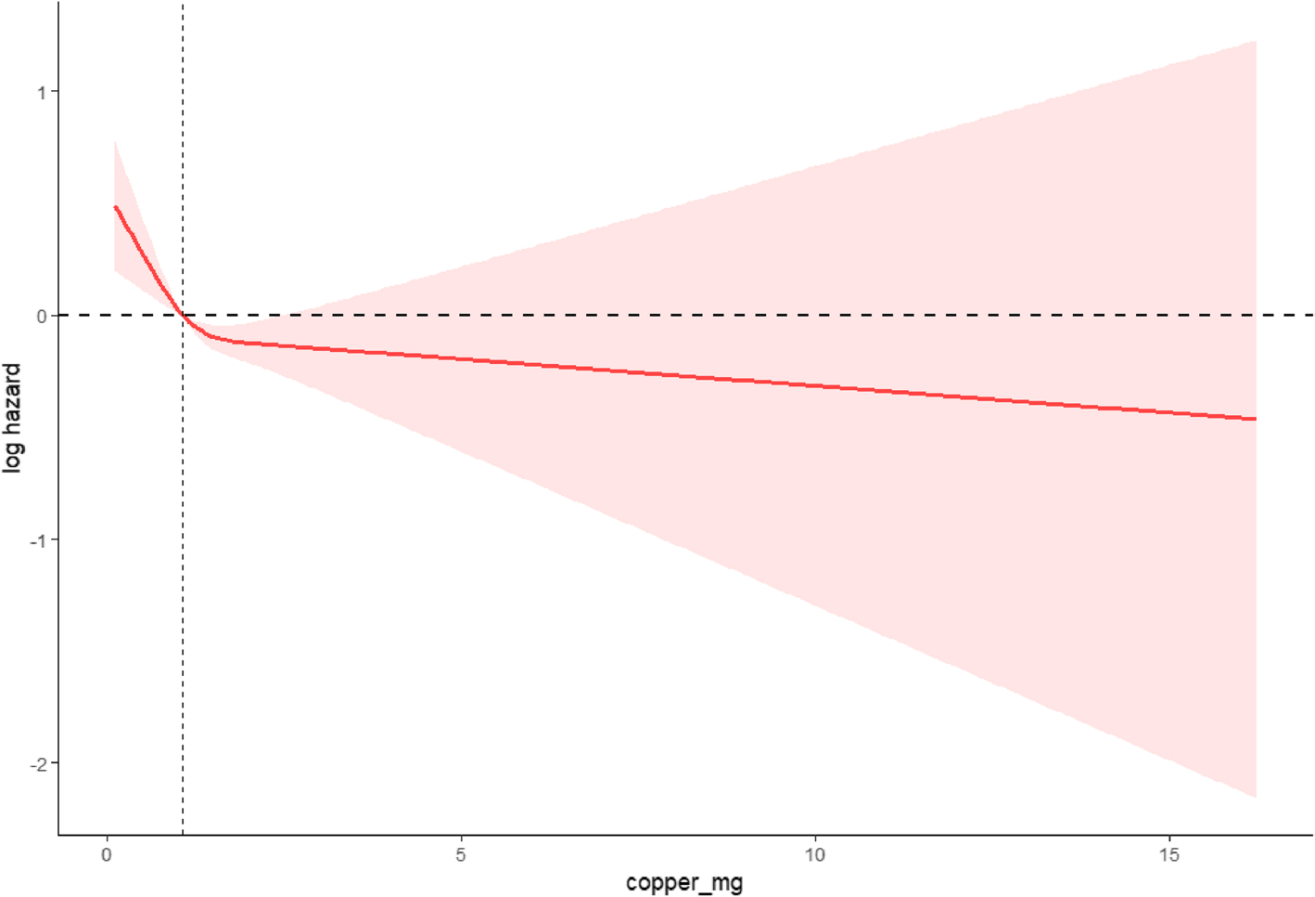
Impact of dietary copper intake on cardiovascular mortality risk in hypertensive patients: a restricted cubic spline analysis

The RCS curve (see Fig. 3) analysis presented here examines the relationship between dietary copper intake and cardiovascular mortality rates. The curve reveals that as copper intake increases from the lowest level, the log hazard of cardiovascular mortality initially decreases rapidly, stabilizing around 5 mg. Beyond this point, even as copper intake continues to increase, the reduction in mortality risk is no longer pronounced, indicating that increasing copper intake is most effective in reducing mortality risk within a low to moderate range, but the marginal benefits diminish once a certain threshold is exceeded. Additionally, the analysis did not detect any significant change points, suggesting that changes in cardiovascular mortality risk across the entire range of intake studied are relatively smooth, without abrupt shifts. These findings suggest that appropriately increasing dietary copper intake may help reduce cardiovascular mortality risk within a certain range but also emphasize the importance of controlling copper intake to avoid excess. This provides crucial scientific support for nutritional recommendations for patients with hypertension.

### Subgroup analysis

In analyzing the relationship between dietary copper intake and cardiovascular mortality among hypertensive patients, significant influences from factors such as gender, ethnicity, education level, and diabetes status were observed. Both males and females benefited from increased copper intake in reducing cardiovascular mortality, with similar effects observed between genders in this table (-0.16, 95% CI -0.26 to -0.07, *P* < 0.001 for males; -0.16, 95% CI -0.23 to -0.10, *P* < 0.0001 for females). This contrasts with the findings in all-cause mortality (Table 5), where gender differences were more pronounced. Among ethnic groups, Whites (-0.16, 95% CI -0.23 to -0.09, *P* < 0.0001) and Mexicans (-0.26, 95% CI -0.39 to -0.13, *P* < 0.001) showed significant decreases in cardiovascular mortality, while no significant reductions were observed in Blacks (*P* = 0.43), possibly due to underlying genetic or environmental factors, or a smaller sample size in this group. Other ethnicities showed a marginally significant reduction (-0.20, 95% CI -0.40 to 0.00, *P* = 0.05). Education level was another critical factor, with individuals holding higher education degrees (college and above) demonstrating stronger protective effects (-0.22, 95% CI -0.40 to -0.05, *P* = 0.01), likely reflecting healthier lifestyles and nutritional habits associated with higher educational attainment. For diabetes status, individuals without diabetes (-0.14, 95% CI -0.22 to -0.06, *P* < 0.001) and those with diabetes (-0.17, 95% CI -0.27 to -0.07, *P* < 0.001) both experienced significant reductions in cardiovascular mortality. Furthermore, a significant trend was observed among individuals with impaired fasting glucose (IFG; -0.16, 95% CI -0.30 to -0.01, *P* = 0.03), while no significant changes were found in those with impaired glucose tolerance (IGT; *P* = 0.40).These findings underscore the importance of dietary copper intake in reducing cardiovascular mortality among hypertensive patients across various demographic and clinical subgroups, with notable variations in effects based on ethnicity, education level, and diabetes status(see table 4).

**Table 4:** Subgroup analysis of the association between dietary copper intake and cardiovascular mortality rate

Character	*95% CI*	*p*	*p for interaction*
**Sex**			1
Male	-0.16(-0.26, -0.07)	< 0.001	
Female	-0.16(-0.23, -0.10)	< 0.0001	
**Ethic**			0.43
White	-0.16(-0.23, -0.09)	< 0.0001	
Other	-0.2(-0.40, 0.00)	0.05	
Black	-0.07(-0.24, 0.11)	0.43	
Mexican	-0.26(-0.39, -0.13)	< 0.001	
**Education**			0.06
Less than 9th grade	-0.14(-0.26, -0.03)	0.01	
9-11th grade (Includes 12th grade with no diploma)	-0.18(-0.35,0.00)	0.05	
High school graduate/GED or equivalent	0.02(-0.07,0.11)	0.70	
Some college or AA degree	-0.14(-0.25, -0.04)	0.01	
College graduate or above	-0.22(-0.40, -0.05)	0.01	
**Diabetes**			0.67
No	-0.14(-0.22, -0.06)	< 0.001	
DM	-0.17(-0.27, -0.07)	< 0.001	
IFG	-0.01(-0.29,0.26)	0.92	
IGT	-0.11(-0.37,0.15)	0.40	
**Alcohol.user**			0.23
Former	-0.19(-0.29, -0.09)	< 0.001	
Mild	-0.12(-0.24,0.00)	0.05	
Heavy	0.08(-0.19,0.34)	0.57	
Moderate	-0.13(-0.30,0.05)	0.15	
Never	-0.17(-0.27, -0.07)	< 0.001	
**Smoke**			0.16
Former	-0.15(-0.24, -0.05)	0.002	
Now	-0.05(-0.22,0.12)	0.55	
Never	-0.22(-0.30, -0.13)	< 0.0001	
**Age**			0.9
< 60	-0.15(-0.21, -0.09)	< 0.0001	
≥ 60	-0.18(-0.83,0.48)	0.45	
**BMI**			0.77
Normal weight	-0.15(-0.31,0.01)	0.06	
Over weight	-0.19(-0.26, -0.11)	< 0.0001	
Obesity	-0.13(-0.23, -0.03)	0.01	

*Abbreviations*: *DM* Diabetes Mellitus, *IFG* Impaired Fasting Glucose, *IGT* Impaired Glucose Tolerance, *BMI* Body Mass Index

For all-cause mortality, gender, ethnicity, education level, diabetes status, and other factors significantly influenced the association between copper intake and mortality reduction. Both men and women benefited from increased copper intake, with significant reductions observed in both groups (-0.17, 95% CI -0.23 to -0.11, *P* < 0.0001 for men; -0.09, 95% CI -0.16 to -0.03, *P* = 0.01 for women). However, the protective effect appeared more pronounced in men, as indicated by the smaller confidence interval and a marginally significant interaction effect (P for interaction = 0.12). Significant reductions in all-cause mortality were observed among Whites (-0.15, 95% CI -0.19 to -0.10, *P* < 0.0001), Mexicans (-0.20, 95% CI -0.31 to -0.08, *P* < 0.001), and other ethnicities (-0.17, 95% CI -0.28 to -0.05, *P* = 0.004). No significant effects were observed in Blacks (*P* = 0.92), which may reflect differences in baseline characteristics or access to care. Higher education levels were associated with stronger protective effects; individuals with some college or AA degrees (-0.11, 95% CI -0.18 to -0.05, *P* < 0.001) and those with 9–11 years of education (-0.19, 95% CI -0.30 to -0.08, *P* < 0.001) demonstrated significant reductions, while individuals with less than 9 years of education or higher degrees (college and above) did not (*P* = 0.13 and *P* = 0.37, respectively). Regarding diabetes status, significant reductions in mortality were observed for individuals with diabetes (-0.13, 95% CI -0.20 to -0.06, *P* < 0.001), without diabetes (-0.11, 95% CI -0.17 to -0.05, *P* < 0.001), and those with impaired fasting glucose (IFG; -0.16, 95% CI -0.30 to -0.01, *P* = 0.03), while no significant effects were found in individuals with impaired glucose tolerance (IGT; *P* = 0.15). Lifestyle factors such as smoking and alcohol consumption also influenced mortality. Former drinkers (-0.12, 95% CI -0.21 to -0.03, *P* = 0.01) and mild drinkers (-0.11, 95% CI -0.18 to -0.03, *P* = 0.01) showed significant reductions in mortality, while heavy drinkers (*P* = 0.85) did not. Similarly, former smokers (-0.16, 95% CI -0.21 to -0.11, P < 0.0001) and never smokers (-0.12, 95% CI -0.21 to -0.02, *P* = 0.01) experienced significant reductions, while current smokers did not (*P* = 0.10). Similarly, age was an influential factor, with individuals under 60 years (-0.13, 95% CI -0.17 to -0.08, *P* < 0.0001) showing significant reductions, while those aged 60 and above did not (*P* = 0.85). These findings underscore the significant protective effects of dietary copper intake on all-cause mortality, with variations across demographic, educational, and clinical subgroups (see table 5).

**Table 5:** Subgroup analysis of the association between dietary copper intake and all-cause mortality rate

Variable	*95% CI*	*P*	*p for interaction*
**Sex**			0.12
Male	-0.17(-0.23, -0.11)	< 0.0001	
Female	-0.09(-0.16, -0.03)	0.01	
**Ethic**			0.08
White	-0.15(-0.19, -0.10)	< 0.0001	
Other	-0.17(-0.28, -0.05)	0.004	
Black	0.01(-0.15,0.17)	0.92	
Mexican	-0.2(-0.31, -0.08)	< 0.001	
**Education**			0.2
Less than 9th grade	-0.08(-0.18,0.02)	0.13	
9-11th grade (Includes 12th grade with no diploma)	-0.19(-0.30, -0.08)	< 0.001	
High school graduate/GED or equivalent	-0.02(-0.10, 0.07)	0.67	
Some college or AA degree	-0.11(-0.18, -0.05)	< 0.001	
College graduate or above	-0.08(-0.25, 0.09)	0.37	
**Diabetes**			0.91
No	-0.11(-0.17, -0.05)	< 0.001	
DM	-0.13(-0.20, -0.06)	< 0.001	
IFG	-0.16(-0.30, -0.01)	0.03	
IGT	-0.16(-0.38, 0.06)	0.15	
**Alcohol.user**			0.55
Former	-0.12(-0.21, -0.03)	0.01	
Mild	-0.11(-0.18, -0.03)	0.01	
Heavy	-0.01(-0.16, 0.13)	0.85	
Never	-0.14(-0.21, -0.07)	< 0.001	
Moderate	-0.14(-0.26, -0.02)	0.03	
**Smoke**			0.38
Former	-0.16(-0.21, -0.11)	< 0.0001	
Now	-0.08(-0.17, 0.02)	0.10	
Never	-0.12(-0.21, -0.02)	0.01	
**Age**			0.55
< 60	-0.13(-0.17, -0.08)	< 0.0001	
≥ 60	0.06(-0.82,0.94)	0.85	
**BMI**			0.22
Normal weight	-0.15(-0.23, -0.06)	0.002	
Over weight	-0.17(-0.23, -0.11)	< 0.0001	
Obesity	-0.09(-0.16, -0.01)	0.02	

## Discussion

This study highlights the critical role of dietary copper intake in cardiovascular health, particularly among hypertensive patients. Our findings demonstrate a nonlinear dose–response relationship, where moderate copper intake (~ 2.85 mg/day) is associated with a reduced risk of cardiovascular morbidity and mortality, but exceeding this level does not confer additional benefits. These results are consistent with previous epidemiological research, which emphasizes the dual nature of trace minerals in cardiovascular health—where both deficiency and excess may have adverse effects [18, 19].

Our findings align with prior studies indicating that both copper deficiency and excess may contribute to cardiovascular risk. Wen et al. reported a negative association between dietary copper intake and myocardial infarction, particularly among individuals with hypertension [18]. Similarly, Bost et al. suggested that copper deficiency is a contributing factor to hypertension and dyslipidemia, both of which are key CVD risk factors [20]. However, our study differs from the PURE-China Study [6], which found a continuous reduction in CVD risk with increasing copper intake, without a plateau effect. Similarly, Li et al. reported a linear reduction in CVD risk with increasing copper intake, without a plateau effect [6]. This discrepancy may be due to differences in baseline dietary patterns, genetic variations in copper metabolism (e.g., ATP7A/ATP7B transporters), or methodological differences in dietary assessment [21].

The cardiovascular effects of copper are primarily mediated through its role in vascular function, oxidative stress regulation, and inflammation. As a cofactor for nitric oxide synthase (NOS), copper is essential for NO production, which promotes vasodilation and blood pressure regulation [6].Copper deficiency has been linked to impaired NO synthesis and increased cardiovascular risk, while excess copper may lead to oxidative stress and endothelial dysfunction [22]. Additionally, copper influences mitochondrial energy metabolism and inflammatory pathways, both of which are involved in cardiovascular disease progression [23] Given these interactions, maintaining optimal copper intake is crucial for cardiovascular health, and further research is needed to clarify the safe upper limit of copper consumption and its long-term effects on cardiovascular outcomes [24].

These findings carry significant clinical implications. Given that both copper deficiency and excess may contribute to cardiovascular risk, it is essential to maintain an optimal dietary intake (~ 2.85 mg/day). Health professionals should prioritize natural food sources such as shellfish, nuts, whole grains, and legumes, while cautioning against excessive supplementation. Additionally, personalized nutrition approaches should be considered, as our subgroup analysis suggests variability in copper’s effects across sex, ethnicity, and metabolic status. Future research should focus on conducting randomized controlled trials (RCTs) to establish causality and clarify the long-term effects of dietary copper on cardiovascular health [6].

Despite these insights, our study has several limitations. First, its observational design prevents definitive causal conclusions, and residual confounding may exist. Second, dietary copper intake was assessed using self-reported 24-h dietary recall, which is prone to recall bias and misclassification errors. Third, NHANES data are cross-sectional and representative of the U.S. population, limiting the external validity of our findings in other populations. Finally, selection bias may have influenced our results, as individuals with complete dietary and health data may not fully represent the broader hypertensive population.

Future research should adopt a multi-faceted approach to further investigate the impact of dietary copper on cardiovascular health, addressing the limitations of previous observational studies. Well-designed prospective cohort studies are needed to validate the long-term effects of moderate copper intake on CVD outcomes, incorporating biomarker-based copper status assessments (e.g., serum copper, ceruloplasmin levels) rather than relying solely on self-reported dietary recall. Additionally, randomized controlled trials (RCTs) should be conducted to establish a causal relationship between copper intake and cardiovascular health by testing dietary copper supplementation among high-risk populations, such as hypertensive patients with low baseline copper levels. These trials should assess key cardiovascular indicators, including blood pressure, lipid profiles, oxidative stress markers (e.g., superoxide dismutase, malondialdehyde), and endothelial function (e.g., flow-mediated dilation, FMD), to elucidate the mechanistic pathways linking copper and CVD risk. Furthermore, genetic and metabolic studies should explore how variations in copper transport genes (e.g., ATP7A, ATP7B) modulate cardiovascular responses to dietary copper intake, as understanding these gene-nutrient interactions could help identify subpopulations that may benefit most from copper interventions. Finally, given the nonlinear relationship observed between copper intake and cardiovascular outcomes, future studies should focus on determining the upper safe intake limit and evaluating the potential risks associated with excessive long-term copper consumption to refine dietary recommendations and public health guidelines.

## Conclusions

This longitudinal study from the NHANES database (2001–2018) has significantly enhanced our understanding of the relationship between dietary copper intake and cardiovascular outcomes in hypertensive patients. We observed that higher copper intake is associated with reduced rates of cardiovascular morbidity and mortality. Specifically, patients with higher copper intake showed lower prevalence and better prognostic outcomes in cardiovascular health compared to those with lower intake levels. These findings suggest that dietary copper may play a crucial role in cardiovascular protection among hypertensive individuals. Therefore, incorporating copper into dietary recommendations could potentially improve cardiovascular health outcomes in this high-risk population. This study supports the need for further research to refine our understanding of copper's benefits and to guide public health strategies aimed at reducing cardiovascular risks through nutrition.

## Data Availability

All data generated or analyzed during this study are included in this published article.
